# TEE Guiding for T-TEER by Using Multiplanar Reconstructions in a Patient With Previous Surgical Annuloplasty

**DOI:** 10.1016/j.jaccas.2024.103180

**Published:** 2025-02-12

**Authors:** Fabian Barbieri, Vasileios Exarchos, Mario Kasner, Ulf Landmesser, Markus Reinthaler

**Affiliations:** a Deutsches Herzzentrum der Charité, Department of Cardiology, Angiology and Intensive Care Medicine, Campus Benjamin Franklin, Berlin, Germany; b Charité–Universitätsmedizin Berlin, corporate member of Freie Universität Berlin and Humboldt-Universität zu Berlin, Department of Cardiology, Berlin, Germany; c Institute of Active Polymers and Berlin-Brandenburg Center for Regenerative Therapies, Helmholtz-Zentrum Hereon, Teltow, Germany; d Berlin Institute of Health at Charité–Universitätsmedizin Berlin, BIH Biomedical Innovation Academy, Berlin, Germany; e DZHK (German Centre for Cardiovascular Research), Partner site Berlin, Berlin, Germany

**Keywords:** interventional echocardiography, multiplanar reconstruction, surgical annuloplasty, TEER, 3-dimensional echocardiography, tricuspid regurgitation, tricuspid transcatheter edge to edge repair

## Abstract

Transesophageal echocardiographic guidance in tricuspid transcatheter edge-to-edge repair may face limitations in imaging, which are sometimes difficult to overcome and lead to an increased risk of periprocedural complications. We present an imaging vignette describing the usefulness of multiplanar reconstructions by 3-dimensional transesophageal echocardiography in a patient with previously implanted surgical tricuspid annuloplasty ring impeding imaging quality. The procedure was conducted successfully by implanting a single device and reducing tricuspid regurgitation from massive to moderate. Multiplanar reconstructions represent a valuable tool with the possibility for improving safety in tricuspid transcatheter edge-to-edge repair by ameliorated visualization of leaflet insertion and grasping.

## Graphical Abstract

**Figure:**
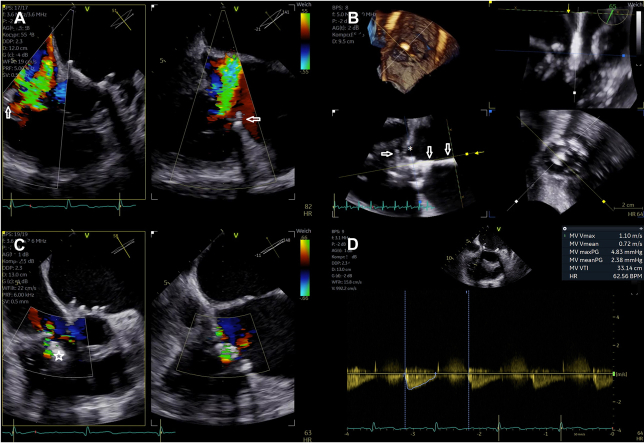

## Case Description

**Figure 1:**
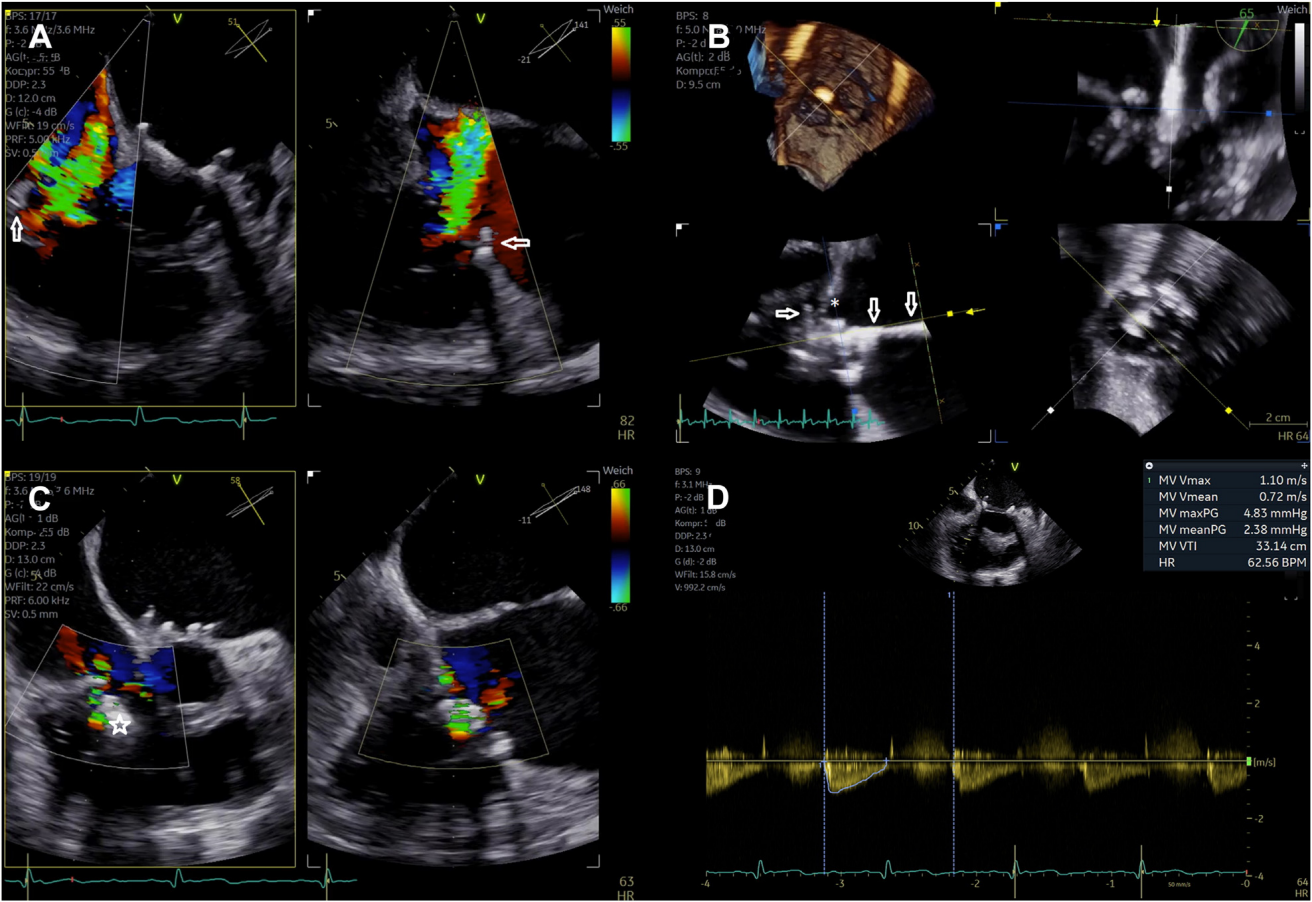
Periprocedural Transesophageal Echocardiographic Guidance (A) Severity of tricuspid regurgitation before performing tricuspid transcatheter edge-to-edge repair (arrows mark the previously implanted annuloplasty ring). (B) Multiplanar reconstruction to visualize the septal leaflet with the delivery system already positioned subvalvularly (arrows, delivery system; asterisk, septal leaflet). Residual tricuspid regurgitation is seen in C (star, transcatheter edge-to-edge-repair device), and (D) confirms adequate postprocedural transvalvular gradient of the tricuspid valve.

With the current increase in tricuspid transcatheter edge-to-edge repair (T-TEER) procedures conducted worldwide, physicians will face a similarly growing number of patients with impaired imaging qualities during transesophageal echocardiographic guidance. We present a successful T-TEER procedure despite a previously implanted surgical annuloplasty ring and associated impaired visibility by using 3-dimensional transesophageal echocardiography and multiplanar reconstruction (Figure 1).

**Take-Home Message**

• Multiplanar reconstruction by 3-dimensional transesophageal echocardiography is a valuable tool to improve procedural safety by allowing excellent alignment of the device to clearly visualize leaflet insertion and grasping.

A 79-year-old woman was admitted for symptomatic congestive heart failure with a history of mitral and tricuspid valve repair by implantation of an annuloplasty ring in 2019. Echocardiographic examination revealed worsening of tricuspid regurgitation (TR) graded as massive (biplane vena contracta 10 × 12 mm, effective regurgitation orifice area 0.6 cm^2^) with a calculated systolic pulmonary artery pressure of 50 mm Hg. During multidisciplinary heart team evaluation, T-TEER was recommended given adequate preprocedural image quality and sufficient leaflet length (Video 1, Video 2, Video 3, Video 4, Video 5). After verbal and written informed consent the procedure was conducted in general anesthesia. This report was approved by the Ethics Committee of Charité–Universitätsmedizin Berlin (EA4/013/21).

With the introduction of the clip delivery system (Pascal, Edwards Lifesciences) through the valve, image quality deteriorated and the leaflets became barely visible (Video 6). Neither adjustments to the settings of the echocardiography machine nor changing position or angulation of the probe led to the desired improvement to safely continue device implantation. As a last resort, 3-dimensional echocardiography and multiplanar reconstructions were utilized. Here, both leaflets were visible enough to verify adequate insertion (Videos 7 and 8). Grasping was then also verified indirectly by closing the device and observing the pulling force (Video 9). Sufficient reduction in TR was achieved without increasing the transvalvular gradient (mean gradient, 2.4 mm Hg). After releasing the device, its position remained stable successfully closing the anteroseptal commissure (Video 10). Assessment of TR showed moderate residual TR (Video 11, Video 12, Video 13, Video 14), which was confirmed by transthoracic echocardiography at discharge (Video 15).

High-quality echocardiographic imaging is a key element for adequate outcomes in T-TEER procedures. Nonetheless, impaired visibility is regularly observed and may hinder safe device deployment. In particular, shadowing owing to structures (eg, calcified aortic valve) or implants (eg, interatrial septal occluder) may aggravate the visibility of leaflets, which increases the risk of inadequate leaflet grasping and consequently the probability of single leaflet device attachments, a dreaded complication with limited possibilities in therapy.^1^^,^^2^ Three-dimensional echocardiography and its possibility to apply multiplanar reconstruction allows excellent alignment of the device to clearly visualize leaflet insertion and grasping. Alternatively, intracardiac echocardiography may be applied to overcome this issue.^3^

## Funding Support and Author Disclosures

The authors acknowledge financial support from the Open Access Publication Fund of Charité–Universitätsmedizin Berlin and the German Research Foundation (DFG). Dr Barbieri has received grant support from Abbott Laboratories and Boston Scientific; has received consulting fees from Boston Scientific and Edwards Lifesciences; and has received speaker honoraria from Edwards Lifesciences. All other authors have reported that they have no relationships relevant to the contents of this paper to disclose.

## Appendix

Video 1 Preprocedural Assessment of Tricuspid Regurgitation Severity in a Biplane Short Axis View

Video 2 Preprocedural Assessment of Leaflet Visualization in a Biplane Short Axis View

Video 3 Preprocedural Assessment of Leaflet Configuration as Well as Space Beneath the Valve to Prevent Chordae Entanglement in a Biplane Transgastric Short Axis View

Video 4 Preprocedural 3-dimensional Visualization of The Tricuspid Valve (En Face)

Video 5 Preprocedural 3-dimensional Visualization of the Tricuspid Valve With Color Doppler (En Face)

Video 6 Impaired Visibility of Leaflets Occurred During Maneuvering in Valve in a Biplane Short Axis View

Video 7 Three-dimensional Transesophageal Multiplanar Reconstruction for Better Visualization of the Leaflets During the Procedure

Video 8 Septal Leaflet Grasping Visualized by 3-dimensional Transesophageal Multiplanar Reconstruction

Video 9 Indirect Visualization of Leaflet Grasping by Inspecting the Closing Force of the Device in a Biplane Transgastric Short Axis View

Video 10 Release of the Device After Verification of Adequate Leaflet Grasping, an Acceptable Transvalvular Gradient, and Significant Reduction of Tricuspid Regurgitation

Video 11 Final Assessment of Tricuspid Regurgitation Severity After Device Release in a Biplane Short Axis View

Video 12 Final Assessment of Tricuspid Regurgitation Severity After Device Release in a Biplane Transgastric Short Axis View

Video 13 Three-dimensional Visualization of the Tricuspid Valve (En Face) After Device Deployment

Video 14 Three-dimensional Visualization of the Tricuspid Valve (En Face) With Color Doppler After Device Deployment

Video 15 Transthoracic Apical Four-chamber View With Color Doppler Verifying Moderate Tricuspid Regurgitation Before Discharge
